# Bovine Rhinitis Viruses Are Common in U.S. Cattle with Bovine Respiratory Disease

**DOI:** 10.1371/journal.pone.0121998

**Published:** 2015-03-19

**Authors:** Ben M. Hause, Emily A. Collin, Joe Anderson, Richard A. Hesse, Gary Anderson

**Affiliations:** Veterinary Diagnostic Laboratory and Department of Diagnostic Medicine and Pathobiology, Kansas State University, Manhattan, Kansas, United States of America; Oklahoma State University, UNITED STATES

## Abstract

Bovine rhinitis viruses (BRV) are established etiological agents of bovine respiratory disease complex however little research into their epidemiology and ecology has been published for several decades. In the U.S., only bovine rhinitis A virus 1 (BRAV1) has been identified while bovine rhinitis A virus 2 (BRAV2) and bovine rhinitis B virus (BRBV) were previously only identified in England and Japan, respectively. Metagenomic sequencing of a nasal swab from a bovine respiratory disease (BRD) diagnostic submission from Kansas identified contigs with approximately 90% nucleotide similarity to BRAV2 and BRBV. A combination of de novo and templated assemblies using reference genomes yielded near complete BRAV2 and BRBV genomes. The near complete genome of bovine rhinitis A virus 1 (BRAV1) was also determined from a historical isolate to enable further molecular epidemiological studies. A 5’-nuclease reverse transcription PCR assay targeting the 3D polymerase gene was designed and used to screen 204 archived BRD clinical specimens. Thirteen (6.4%) were positive. Metagenomic sequencing of six positive samples identified mixed BRAV1/BRAV2, BRAV1/BRBV and BRAV2/BRBV infections for five samples. One sample showed infection only with BRAV1. Seroprevalence studies using a cell culture adapted BRBV found immunofluorescence assay-reactive antibodies were common in the herds analyzed. Altogether, these results demonstrate that BRV infections are common in cattle with respiratory disease and that BRAV1, BRAV2 and BRBV co-circulate in U.S. cattle and have high similarity to viruses isolated more than 30 years ago from diverse locations.

## Introduction

Along with equine rhinitis virus (ERV) and foot and mouth disease virus (FMDV), bovine rhinitis A and B viruses (BRAV and BRBV, respectively) are species in the genus *Aphthovirus*, family *Picornaviridae* [1]. Two serotypes of BRAV have been identified, BRAV1 and BRAV2, while BRBV consists of a single serotype. The BRAV1 strain SD-1 was isolated in Germany in 1962 from nasal secretions from a calf with rhinitis [2]. Additional BRAV1 strains were subsequently isolated from both healthy and diseased bovines in England, Japan, Italy and the U.S. and shown to cross react in serum neutralization assays [3–6]. The sole BRBV isolate EC-11 was isolated in England in 1964 by Reed from the lung of a specific pathogen free calf with respiratory disease [7]. Likewise, BRAV2 consists of a single specimen, strain H-1, isolated from an outbreak of respiratory disease in cattle in 1984 [8]. Despite numerous studies on bovine rhinitis viruses (BRV) in the 1960’s through mid-1980’s, little work has been published on their epidemiology and ecology the past several decades.

Bovine respiratory disease complex (BRDC) is the most economically significant disease of the cattle industry, leading to losses due to mortality, morbidity, treatment costs and feed inefficiency in excess of $750 million dollars per year in the U.S. alone [9]. BRDC has a multifactorial etiology involving a variety of bacteria and viruses in addition to host and environmental factors [10]. Numerous commercial vaccines including both killed and attenuated live bacteria are available. Viruses commonly included in commercial vaccine include bovine viral diarrhea virus (BVDV), bovine herpes virus 1 (BHV1), parainfluenza virus 3 (PI3) and bovine respiratory syncytial virus (BRSV). Despite their widespread use, BRDC incidence has increased over the past 20 years [11,12]. BRDC pathogenesis often involves a primary viral infection which damages respiratory mucosa and alters host immune responses leading to secondary bacterial pneumonia caused by commensal bacteria already present in the respiratory tract [13].

Both BRAV and BRBV are established but rarely studied etiologic agents of BRDC. Experimental inoculation of calves with BRAV1 via intranasal (IN) or intratracheal (IT) routes, either singly or in combination, resulted in variable clinical signs of respiratory disease and histologic lesions consistent with pneumonia [14]. BRAV1 was also recovered from nasal swabs of IN inoculated animals and all animals inoculated or exposed by contact seroconverted to BRAV1 by day seven post inoculation. A similar experiment using a different BRAV1 strain (RS 3x) and colostrum deprived calves failed to reproduce clinical disease but was successful in isolating BRAV1 from nasal swabs post inoculation and found histological lesions of focal rhinitis and a neutralizing antibody response in all inoculated calves [15]. BRBV pathogenesis was investigated using intranasal inoculation of gnotobiotic calves [16]. Clinical signs including fever, nasal discharge and increased respiration rate were observed. Foci of epithelial necrosis were observed histologically in the turbinates and trachea and interstitial pneumonia was evident in the lungs. Virus was isolated from multiple tissues and was neutralized by convalescent antiserum. In addition to controlled studies, numerous investigations of acute respiratory disease in cattle resulted in the isolation of bovine rhinitis viruses where paired acute and convalescent sera suggested a causative role for bovine rhinitis virus [6,8,17,18].

Metagenomic sequencing on nasal swabs obtained from BRDC diagnostic submissions were performed to survey viruses present. Contigs with high identity to BRAV2 and BRBV were identified in one swab. To further our understanding of the epidemiology and ecology of bovine rhinitis viruses in BRDC, a more comprehensive survey was performed.

## Materials and Methods

### Ethics Statement

Bovine clinical samples used in this study were submitted to KSVDL for routine diagnostic testing. The samples were obtained from naturally infected animals in the field by licensed veterinarians as a part of normal veterinary care and diagnostic investigations.

### Molecular screening of bovine viruses

Clinical samples (nasal and pharyngeal swabs and lung tissue) from bovine respiratory disease submissions to KSVDL were screened by a BRDC PCR panel which detects BVDV, BHV-1, BRSV and bovine coronavirus (BCV) [19]. A total of 204 samples were screened. Samples were collected in years 2011–2014 from twelve states: Nebraska (n = 48), Kansas (n = 112), Colorado (n = 6), Missouri (n = 1), Mississippi (n = 7), Texas (n = 4), Oklahoma (n = 2), Idaho (n = 2), Montana (n = 6), Oregon (n = 4), Washington (n = 11) and Virginia (n = 1). Samples were also screened for influenza D virus (IDV) using quantitative real time reverse transcription PCR as previously described [20]. A 5’-nuclease reverse transcription PCR assay was designed to detect bovine rhinitis viruses targeting the 3D polymerase gene: probe, 5’-FAM-CGG CAG TCC AGG TCC AGT GT-Iowa Black-3’; Forward: 5’-CTT TTC GGT GTG ATT GGC AG-3’; Reverse: 5’-GAA ATC TAT CAG GGC AGG TCT G-3’. Viral RNA was extracted using the MagMAX-96 viral RNA isolation kit (Life Technologies) according to the manufacturer’s instructions. Real time reverse transcription PCR was performed using Qiagen Quantitect RT-PCR with BRV primers and probe as follows: 50°C, 30 minutes; 95°C, 15 minutes; followed by 40 cycles of 94°C for 15 seconds and 60°C for 60 seconds. The PCR assay specificity was confirmed using bovine rhinitis virus positive samples as determined by metagenomic sequencing as well as with cultures of common BRDC pathogens BVDV, BHV-1, BRSV, BCV, *Mannheimia haemolytica*, *Histophilus somni*, *Pasteurella multocida* and *Mycoplasma bovis*.

### Metagenomic Sequencing

Sample preparation for metagenomic sequencing was performed similar to previously described [21]. In brief, swabs or tissue homogenate were clarified by centrifugation followed by filtration through a 0.2 micron syringe filter. Samples (180 μl) were treated with a cocktail of nucleases to degrade host or unprotected environmental nucleic acids at 37°C for 90 minutes. Viral nucleic acids were isolated using the MinElute Virus spin filter kit (Qiagen) according to the manufacturer’s instructions. Reverse transcription was performed using primers consisting of a known 20 nt sequence followed by (N)_6_ at the 3’ end [22] using a Superscript III reverse transcription kit (Life Technologies). Second strand synthesis was performed using Sequenase 2.0 (Affymetrix). Double stranded cDNA was purified using a Qiagen Minelute PCR spin column and subsequently amplified using primers identical to the known 20bp region of the random hexamer containing primer. Amplicons were purified using a Qiagen Minelute PCR spin column and quantified using a Qubit fluorimeter (Life Technologies). Samples were diluted to 0.2 ng/μl and 5μL was used for sequencing library preparation using the Nextera XT library preparation kit (Illumina) according to manufacturer’s instructions. Pooled barcoded libraries were sequenced on an Illumina MiSeq instrument using paired 150bp reads.

Reads from each sequencing library were parsed into individual folders based on barcoded sequences. Reads were imported into the CLC Genomics software package (Qiagen). Reads were mapped to the host genome (*Bos taurus*) and unmapped reads were collected. De novo assembly was performed on unmapped reads and assembled contigs were analyzed by BLASTN. BRV genomes were assembled using a combination of de novo and templated assembly with reference BRAV2 and BRBV genomes (GenBank JN936206 and EU236594, respectively). Raw sequencing reads were submitted to the National Center for Biotechnology Information Sequence Read Archive under BioProject PRJNA273738 (accession number SRP052864). Accession numbers for the individual samples are SRR1776513 (sample 140032–4), SRR1776531 (sample 5–52), SRR1776533 (sample 6–17), SRR1776541 (sample 20–76), SRR1776564 (sample 21–22), SRR1776524 (sample 4–26) and SRR1776537 (sample 6–19). The genomes for BRAV1 Sd-1, BRAV2 140032–1 and BRBV 140032–2 were submitted to GenBank under accessions KP236128, KP236129 and KP236130, respectively. Phylogenetic analyses were performed by using Mega6.06 software using the Maximum Likelihood algorithm with tree topology verified by performing 1000 bootstrap replicates [23].

### Cells and Viruses

Fetal bovine kidneys, sourced from an abattoir, were purchased from Innovative Research. Primary embryonic bovine kidney (EBK) cells were cultured by treatment of finely minced kidneys with 0.25% trypsin at 37°C for 4 hours following transfer to cell culture flasks with MEM with L-glutamine and 100 units/mL penicillin, 100 μg/mL streptomycin and 0.25 μg/mL amphotericin B. Madin-Darby bovine kidney (MDBK) (ATCC CCL-22) were maintained in MEM with L-glutamine and 5% fetal calf serum at 37°C with 5% CO_2_. BRBV (ATCCVR-1806) was first passaged on EBKs before being expanded on MDBK cells. Virus was grown in MEM at 33°C with 5% CO_2_.

### Indirect immunofluorescence assay

Indirect immunofluorescence was performed using 59 bovine serum samples submitted for diagnostic testing at KSVDL. The samples were originated from Kansas (n = 36), Illinois (n = 5), Missouri (n = 9), Texas (n = 2), Colorado (n = 2) and Idaho (n = 5) from beef and dairy herds. Antiserum from a gnotobiotic calf hyperimmunized with BCV was used as a negative control (National Veterinary Services Laboratories catalog #320-BDV). Sera from two high health cows housed in isolation at the Kansas State University Veterinary Health Center were also used as negative controls. Non-infected cells were also stained to determine level of background fluorescence. MDBK cells were infected with BRBV at a dilution of 1:15 and incubated overnight. Once the onset of cytopathic effect (CPE) was evident in approximately 20% of the cells, plates were washed with PBS and fixed in 80% aqueous acetone. Serum was added at a 1:10 dilution and seven serial two-fold dilutions were made in PBS, with a final serum dilution of 640. Plates were incubated for 1 hour at 37°C, then washed three times with PBS. FITC-conjugated rabbit anti-bovine IgG (Jackson Immunoresearch) was added at a dilution to 1:50, along with Evan’s Blue at 1:500, and incubated for 1 hour at 37°C. Plates were then washed three times with PBS and 100 μL of 50% glycerol in PBS was added to each well and subsequently read on a fluorescence microscope.

## Results and Discussion

### Metagenomic Sequencing of Clinical Sample

In September, 2013, nasal swabs were submitted to the Kansas State Veterinary Diagnostic Laboratory (KSVDL) from an approximately 6-month old calf in Kansas with acute respiratory disease. Aerobic culture identified *Mannheimia haemolytica*. A bovine respiratory disease PCR panel was also performed and was positive for *Mycoplasma bovis* [cycle threshold (C_t_) = 27.8] and bovine coronavirus (BCV, Ct = 20.0). Metagenomic sequencing was next performed on the sample to investigate BCV genetic diversity for an unrelated project. The nasal swab sample was filtered through a 0.2 micron syringe filter and treated with a cocktail of nucleases before nucleic acid isolation. Reverse transcription, second strand synthesis and amplification were performed using sequence independent single primer amplification (SISPA) and sequencing libraries were prepared from the resulting amplicons [22]. Approximately 4 million reads were generated on an Illumina MiSeq instrument. Reads mapping to host DNA were subtracted (∼3.6M reads) and the remaining sequences (∼430k reads) were assembled *de novo* into 192 contigs and classified based on best BLASTN expectation (*E*) scores. Besides the expected BCV, contigs with similarity to bovine adenovirus 4 (BAdV4), BRAV2 and BRBV were identified with *E* values of 0. Templated assembly was next performed using reference genomes identified by BLAST analysis. A near complete BCV genome was assembled from 15,500 reads to yield a consensus sequence of 31,017 nucleotides (nt) (missing the 5’-terminal 4 nt) with 99% identity to strain E-AH65 [24]. Significantly fewer reads mapped to BAdV4 (238 reads) which resulted in ∼25% genome coverage with 91% identity to strain THT/62 [25]. Assembly using a BRAV2 reference genome mapped 5,924 reads which spanned the 5’-non coding region and the complete polyprotein open reading frame with the exception of a 16 nt gap approximately 150 nt from the 3’-end of the polyprotein. The determined BRAV2 sequence was 89% identical to the BRAV2 strain H-1. Similarly, assembly using the BRBV reference genome identified 2,505 reads which spanned the entire reference sequence with the exception of 48 and 13 nt at the 5’ and 3’-termini. The consensus sequence was 87% identical to BRBV strain EC11 [1]. As only a single genome each of BRAV2 and BRBV have been published and neither of the viruses have previously been identified in U.S. cattle, further investigation was warranted.

### Genome Sequence BRAV1 strain Sd-1

There are two serotypes of BRAV, 1 and 2. While the genome sequence of BRAV2 strain H-1 is available in GenBank, no sequence for BRAV1 is available. To enable further molecular epidemiology studies on bovine rhinitis viruses, the genome of BRAV1 strain Sd-1 was determined. BRAV1 Sd-1 was obtained from American Type Culture Collection (ATCC) and sequenced using metagenomic sequencing methodology as described above directly from cell culture harvests of BRAV1 Sd-1 obtained from ATCC. Genome assembly was performed using a combination of de novo and templated assemblies using the BRAV2 strain H-1 reference. A 7,245 bp contig with 82% identity to BRAV2 H-1 was determined from ∼200,000 reads mapping to BRAV2 spanning the entire BRAV2 H-1 reference sequence with the exception of the 5’-terminal 4 nt.

### BRAV1 strain Sd-1 Sequence Analysis

Similar to all picornaviruses, the genome of BRAV1 consists of a long 5’ non-translated region (NTR), a single large ORF encoding a polyprotein and a short 3’-NTR followed by a poly-A tail. The region of the 5’ NTR sequenced encompasses 537 nt and is 89% identical to BRAV2 H-1. The 5’ NTR contained three insertions (G334, C350, C527) and one deletion (A345) as compared to BRAV2 H1. The Aphthovirus 5’NTR contains a type II internal ribosome entry site [26–28]. The polyprotein initiation codon is typically preceded by a polypyrimidine tract by approximately 20 nt’s. A polypyrimidine tract is present in BRAV1 from nt 514–522 (UUUUCCUUU) followed by an AUG initiation codon at nt 538–540. A second initiation codon is present closely downstream and in the same reading frame at nt 553–555. Aphthoviruses typically express two different forms of the leader polypeptide (Lab and Lb) from alternate initiation codons [29–30]. FMDV favors translation from the second initiation codon while ERAV favors the first AUG. AUG codons favored for translation are located in the context of Kozak sequences [31]. For BRAV1, the second AUG from nt 553–555 is in the context of a strong Kozak sequence and expression of a 2,213 amino acid polyprotein is expected to be dominant. The complete ORF of BRAV1 Sd1 encodes a predicted 2,218 amino acid polypeptide with 93% identity to BRAV2 and 50% identity to BRBV (Table 1). The polyprotein is processed by viral proteases into L, four structural (VP4, VP2, VP3, VP1) and seven non-structural (2A, 2B, 2C, 3A, 3B, 3C, 3D) proteins. The non-structural proteins of BRAV1 were very similar to those of BRAV2 with over 90% identity. The most divergent proteins were the capsid proteins VP1, VP2 and VP3 with 84.9–87.5% identity. VP1, VP2 and VP3 are the only surface-exposed viral proteins and consequently are antigenic determinants [32,33]. As BRAV1 and BRAV2 are serologically distinct, the sequence divergence observed between VP1, VP2 and VP3 represents serotypic differences [8].

**Table 1:** Amino acid length and sequence identity (%) between BRAV1 strain Sd-1 and other bovine rhinitis viruses.

Region	Protein Length (aa)	% Identity BRAV2 H-1	% Identity BRAV2 140032–1	% Identity BRBV EC-11	% Identity BRBV 140032–2
L	179	92.2	82.4	39.9	39.4
VP4	93	94.6	95.7	64.9	66.0
VP2	224	86.2	87.5	43.2	43.2
VP3	221	85.5	86.0	55.4	52.3
VP1	232	84.9	85.3	36.4	36.0
2A	20	90.0	95.0	35.9	35.9
2B	127	98.4	98.4	39.9	40.6
2C	311	97.4	98.1	59.6	59.6
3A	109	95.4	94.5	27.1	27.1
3B	24	95.8	100.0	50.0	50.0
3C	209	97.6	97.1	58.6	57.6
3D	469	97.9	98.1	58.9	58.6
Total	2218	93.2	92.7	50.4	50.1

### BRAV2 strain 140032–1 Sequence Analysis

The region of the 5’NTR of BRAV2 strain 140032–1 sequenced (487 nt) was 90% identical to BRAV2 strain H-1 (Table 2). The 5’NTR contained four deletions (T195, T197, C343, A345) and four insertions (T198, G199, C339, C341). A large ORF was identified from nt 488–7,219 encoding a predicted 2,243 amino acid polypeptide with 97% identity to BRAV2 H-1 that was preceded by a polypyrimidine tract of CCCCUCUUU at nt 468–476. The L protein of BRAV2 strain 140032–1 was unusual such that it had four initiation codons in frame with the polypeptide within the first 100 bp of the ORF (nt’s 488–490, 539–541, 554–556 and 578–580). The latter initiation codon at 578–580 however was the only one associated with a Kozak sequence. Further experimentation is required to determine which initiation codon(s) is used by BRAV2. The L protein initiated at nt 488 is predicted to encode 204 amino acids and has only 82.4 and 82.8% identity to BRAV1 and BRAV2 strain H-1, respectively, and is the most divergent protein of 140032–1. The Lab proteins of BRAV1 and BRAV2 strain H-1 consist of 179 amino acids while the full length L protein of 140032–1 is more similar in length to that of BRBV (207 amino acids). The L protein autocatalytically cleaves the polyprotein at the L/P1 junction and also catalyzes the cleavage of the p220 subunit of the cap binding complex eIF4G resulting in inhibition of cellular cap-dependent translation initiation [34]. Further studies are required to determine if translation initiation of 140032–1 occurs at all four codons and if these proteins have similar biological properties. The structural and non-structural proteins of 140032–1 all had >95% identity to BRAV2 H-1.

**Table 2:** Amino acid length and sequence identity (%) between BRAV2 strain 140032–1 and other bovine rhinitis viruses.

Region	Protein Length (aa)	% Identity BRAV1 Sd-1	% Identity BRAV2 H-1	% Identity BRBV EC-11	% identity BRBV 140032–2
L	204	82.4	82.8	41.4	40.9
VP4	93	95.7	97.9	64.9	66.0
VP2	224	87.5	98.2	43.7	43.2
VP3	221	86.0	97.7	51.8	50.5
VP1	232	85.3	97.4	36.4	37.7
2A	20	95.0	95.0	35.9	35.9
2B	127	98.4	100.0	39.1	39.9
2C	311	98.1	98.7	58.7	58.7
3A	109	94.5	97.3	27.1	27.1
3B	24	100.0	95.8	50.0	50.0
3C	209	97.1	99.5	58.1	57.1
3D	469	98.1	99.2	58.9	58.9
Total	2243	92.7	97.0	50.1	50.0

### BRBV strain 140032–2 Sequence Analysis

A 566 nt region of the 5’NTR was sequenced for 140032–2 that was 93% identical to BRBV strain EC-11 (Table 3). The 5’ NTR had a single insertion of a C at position 59 and a single deletion of C529. A large ORF from nt 567–7,409 was identified that codes for a predicted 2,280 amino acid polypeptide with 94.6% identity to EC-11. Similar to EC-11, two initiation codons in frame with the polypeptide were identified near the 5’-terminus (nt’s 567–569 and 639–641). Translation from the latter codon beginning at nt 639 is predicted to be more favorable as it is in the context of a Kozak sequence. With the exception of proteins VP1 and 2A, all proteins had greater than 92% identity to EC-11. Interestingly, VP1 showed only 86.8% amino acid identity to EC-11. VP1 is exposed on the virion surface and at least three antigenic regions are targeted by neutralizing antibodies [35]. As VP1 from the two serotypes of BRAV share approximately 85% amino acid identity, 140032–2 may represent a new serotype of BRBV however serological experiments are required to confirm this hypothesis.

**Table 3:** Amino acid length and sequence identity (%) of BRBV strain 130024–2 and other bovine rhinitis viruses.

BRBV 140032–2	Protein Length (aa)	% Identity BRAV1 Sd-1	% Identity BRAV2 H-1	% Identity BRAV2 140032–2	% Identity BRBV EC-11
L	207	39.4	38.9	40.9	92.8
VP4	92	66.0	67.0	66.0	96.7
VP2	228	43.2	42.8	43.2	93.0
VP3	219	52.3	50.9	50.5	93.6
VP1	219	36.0	36.9	37.7	86.8
2A	39	35.9	35.9	35.9	87.2
2B	127	40.6	39.9	39.9	94.5
2C	316	59.6	59.0	58.7	100.0
3A	133	27.1	27.1	26.3	92.5
3B	25	50.0	50.0	46.2	100.0
3C	208	57.6	57.6	57.1	98.1
3D	467	58.6	58.4	58.9	95.7
Total	2280	50.1	49.9	50.0	94.6

### Phylogenetic Analysis

**Fig 1:**
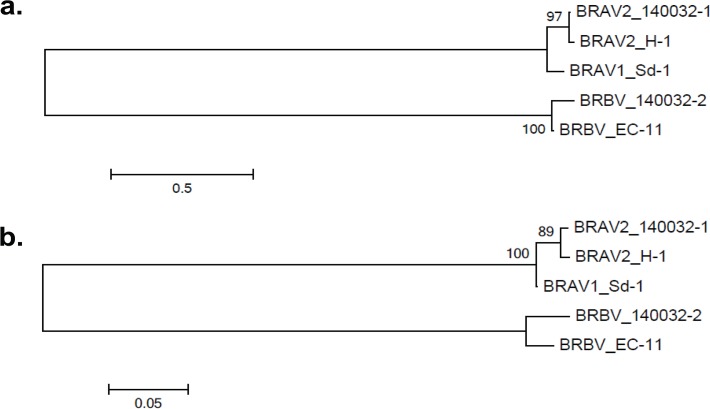
Phylogenetic trees of the coding regions of a) P1 (VP4, VP2, VP3, VP1) and b) 3D polymerase. Maximum-likelihood analysis in combination with 1000 bootstrap replicates was used to derive trees based on the predicted protein sequences. A scale representing the number of amino acid changes is shown in each panel.

To determine the evolutionary relationship between BRAV1, BRAV2 and BRBV, phylogenetic analysis was performed on the P1 protein encoding capsid proteins VP1–4, as well as the highly conserved 3D polymerase protein (Fig. 1). Analysis of P1 found that BRAV2 140032–1 and BRBV 140032–2 formed well-defined clades with BRAV2 H-1 and BRBV EC-11, respectively, while BRAV1 Sd-1 represented as an outlier most closely related to BRAV2. Phylogenetic analysis of 3D polymerase also found well-defined clades for BRBV and BRAV2 with BRAV1 as an outlier closely related to BRAV2.

### Molecular Epidemiology

As metagenomic sequencing of a nasal swab from a calf with acute respiratory disease identified concurrent infection with two bovine rhinitis virus species, we designed a 5’-nuclease reverse transcription PCR (rtPCR) assay targeting the conserved 3D polymerase gene to investigate the incidence of BRV in BRDC diagnostic submissions. A subset of six samples positive on this assay were further confirmed as BRV by metagenomic sequencing (below). Assay specificity was verified using pure cultures of common BRDC pathogens; all were negative. Clinical samples from bovine respiratory disease submissions to KSVDL were previously screened by a BRDC PCR panel which detects BVDV, BHV-1, BRSV and BCV [19]. Samples were separately analyzed for IDV using a previously published method [20]. A total of 204 samples were screened. Thirteen of 204 samples (6.4%) were positive for BRV with Ct values 18.0–29.4. Positive samples originated from Kansas (n = 12) and Nebraska (n = 1). Nine samples were positive for BRV in addition to at least one other virus tested. Four samples were only positive for BRV. Incidences for other viruses involved in BRDC pathogenesis were 36% for BCV (n = 75), 4.8% for IDV (n = 10), 7% for BHV1 (n = 15), 5% for BVDV (n = 11) and 3% for BRSV (n = 7). These results demonstrate that BRV are present in BRDC clinical samples with a similar incidence to other established BRD etiological agents.

To further characterize a subset of the BRV positive samples, six samples were analyzed by metagenomic sequencing in addition to the original sample 140032 from which BRAV2 and BRBV genomes were assembled. Following subtraction of reads mapping to the host genome, sequences were assembled de novo and the resulting contigs were analyzed by BLASTN against a database of the BRV genomes (BRAV1 Sd-1, BRAV2 H-1, BRAV2 140032–1, BRBV EC-11, BRBV 140032–2). An *E* value of ≤1x10^–5^ is generally considered significant for organism identification by metagenomic sequencing [36]. One sample, 6–17, had four contigs with best blast hits only to BRAV1 (Table 4, *E* values 8.9x10^–78^–6.1x10^–146^). Four samples, 5–52, 6–19, 20–76 and 21–22, showed evidence of co-infection with two different BRV (BRAV1 and BRAV2, BRAV1 and BRBV, BRAV2 and BRBV). Sample 4–26 had multiple individual contigs with high similarity to all three species/serotypes. Resequencing of 140032 found contigs mapping to BRAV2 and BRBV similar to the initial analysis. Likewise, reanalysis of the raw data obtained for the reference BRAV1 Sd-1 found contigs with BLASTN hits solely to BRAV1. Similar analysis of metagenomic sequences from a BRV PCR-negative nasal swab submitted for BRD diagnostic testing failed to identify any contigs with *E* values >10^–5^ to BRV. Together, these results suggest that co-infections with multiple species and/or serotypes of BRV are common in cattle. Similar observations were made during the discovery of BRBV, where analysis of viral isolates from cattle with acute respiratory disease found 32 out of 38 isolated viruses were BRAV1 using serum neutralization tests [8]. The remaining six isolates were not neutralized by BRAV1 or BRBV antiserum and were subsequently shown to represent a new serotype, BRAV2.

**Table 4:** BLASTN results from de novo assembled metagenomic sequences of bovine rhinitis virus PCR-positive samples.

Sample	Virus	Contigs	*E* value range	Other viruses-NGS^a^	Other viruses-RT-PCR^b^
4–26	BRAV1	3	3.1x10^−64^–5.7x10^−120^	None	BRV (22.7), BCV (30.6)
	BRAV2	2	5.4x10^−120^–0.0		
	BRBV	10	3.6x10^−43^–0.0		
5–52	BRAV1	2	1.1x10^−56^–3.01x10^−124^	None	BRV (22.6)
	BRAV2	5	7.2x10^−9^–0.0		
6–17	BRAV1	4	8.8x10^−78^–6.1x10^−146^	BPI3 (0.0)	BRV (26.5)
6–19	BRAV1	2	5.5x10^−68^–7.4x10^−73^	None	BRV (27.4), BCV (35.1)
	BRBV	7	2.9x10^−110^–0.0		
20–76	BRAV1	2	9.8x10^−92^–0.0	None	BRV (22.5), BHV1 (34.0)
	BRAV2	1	0.0		
21–22	BRAV1	3	1.9x10^−66^–0.0	BCV (0.0), BRSV (8.5x10^−55^)	BRV (18.8), BRSV (27.8), BCV (25.3), IDV (33.5)
	BRAV2	7	1.3x10^−74^–0.0		
140032	BRAV2	7	8.4x10^−48^–0.0	BAdV4 (0.0), BCV (0.0)	BCV (20.0)
	BRBV	16	7.8x10^−52^–0.0		
BRAV Sd-1	BRAV1	2	0.0	None	n.d.^c^

^a^Viruses identified by BLASTN analysis of contigs assembled de novo. *E* values are in parentheses.

^b^Viruses detected by the RT-PCR. Viruses screened by RT-PCR include BVDV, BCV, BRSV, BRV, BHV1 and IDV. Cycle threshold values are in parentheses.

^c^not determined

The six BRV-positive samples were also analyzed by BLASTN at the National Center for Biotechnology Information using the nucleotide collection database. Reads mapping to the host *Bos Taurus* were subtracted and the remaining reads were assembled de novo and the resulting contigs were analyzed by BLASTN. Contigs with similarity to BRV were identified for all six samples. No other bovine viruses were identified in four of the samples (4–26, 5–52, 6–19 and 20–76; Table 4). Bovine parainfluenza virus 3 (BPI3) was identified in sample 6–17 (*E* = 0.0). BLASTN analysis of 21–22 identified BCV (E = 0.0) and BRSV (E = 8.5x10^–55^). Analysis of 140032 identified bovine adenovirus 4 (BAdV4, *E* = 0.0) and BCV (*E* = 0.0). Viruses identified by metagenomic sequencing were in good agreement for those included in the BRDC RT-PCR panel where Ct values were less than 30: for 21–22, both metagenomic sequencing and RT-PCR identified BCV and BRSV; for 140032, both assays detected BCV. The inability for metagenomic sequencing to detect viruses with Ct values greater than 30 is likely due to insufficient sensitivity.

### Seroprevalence

Indirect immunofluorescence assays were performed on a collection of bovine sera using cell culture adapted BRBV to determine seroprevalence. Sera represented 15 herds in six states. All samples were positive with a titer of 160-≥640. Where age of animal was known (n = 44), the minimum age was 9 months (n = 4) and the majority of animals were adults >2 years of age. Controls including gnotobiotic calf antiserum, sera from high health animals held in isolation and uninfected cells were negative. Attempts to perform IFA using BRAV1 and primary embryonic bovine kidney cells were unsuccessful due to non-specific fluorescence. IFA was not performed with BRAV2 as we were unable to acquire the virus. These results suggest that BRBV infections are common in cattle however additional serological testing is needed to confirm these results. In particular, while BRAV and BRBV are serologically distinct from one another in serum neutralization assays, cross reactivity between BRAV and BRBV in our IFA assay was not performed [7]. Similar detection rates and seroprevalence were recently reported for the newly described bovine IDV [37]. IDV was found in 4.8% of BRD diagnostic submissions and serosurveys found 87.5% of herds and individuals had hemagglutination inhibition assay titers greater than 40. Previous serological studies also found evidence for widespread BRV infections [4,7,17,38,39].

While BRV are established etiological agents for BRDC, little work has been published on these viruses over the past several decades. This is likely in part due to difficulty in isolating these viruses in cell culture. Nearly all reports of BRV isolation utilized primary cells and the titers obtained in vitro were relatively low. Also, controlled animal inoculation with BRV resulted in mild respiratory disease and histopathology. Combined, these results likely contribute to the lack of research on these viruses. Advances in sequencing technology now allow researchers to readily characterize metagenomic samples in a sequence-independent manner, enabling detection and characterization of both established and novel or neglected organisms.

The serological evidence for BRBV and molecular data for bovine rhinitis viruses herein suggest that BRV infections are common components of BRDC. These results, combined with previous controlled inoculation studies for BRAV1 and BRBV, suggest BRV are pathogens involved in BRDC. While our results do not demonstrate a definitive role for BRV in BRDC pathogenesis, we were unable to identify any other viruses present in over half of BRV-positive BRDC clinical samples using metagenomic sequencing. Further research is needed to differentiate the possibility of BRV being a non-pathogenic commensal organism. This first report of BRAV2 and BRBV in U.S. cattle and the occurrence of concurrent mixed BRV co-infections further expands our knowledge on the epidemiology of these viruses.

## References

[1] Hollister JR, Vagnozzi A, Knowles NJ, Rieder E. Molecular and phylogenetic analyses of bovine rhinovirus type 2 shows it is closely related to foot-and-mouth disease virus. Virology 2008;373: 411–425. 10.1016/j.virol.2007.12.019 18201745

[2] Bögel K, Bohm H. Ein rhinovirus des rindes. Zentralbl Bakteriol Orig. 1962;193: 2–14.

[3] Ide PR, Darbyshire JH. Rhinoviruses of bovine origin. Br Vet J. 1969;125: 7–8. 4305981 10.1016/s0007-1935(17)49169-x

[4] Mohanty SB, Lillie MG. Isolation of a bovine rhinovirus. Proc Soc Exp Biol Med. 1968;128: 850–852. 4299210 10.3181/00379727-128-33140

[5] Persechi A. Isolation and characterization of rhinoviruses from calves. Acta Med Vet. 1974;20: 333–358.

[6] Shimizu Y, Narita M, Murase N. Isolation of a bovine rhinovirus from calves with respiratory disease. Natl Inst Anim Health Q. 1974;14: 35–41. 4374662

[7] Reed SE, Tyrrell DA, Betts AO, Watt RG. Studies on a rhinovirus (EC11) derived from a calf. I. Isolation in calf tracheal organ cultures and characterization of the virus. J Comp Pathol. 1971;81: 33–40. 4326295 10.1016/0021-9975(71)90052-1

[8] Yamashita H, Akashi H, Inaba Y. Isolation of a new serotype of bovine rhinovirus from cattle. Arch Virol. 1985;83: 113–16. 2982346 10.1007/BF01310969

[9] Griffin D. Economic impact associated with respiratory disease in beef cattle. Vet Clin North Am Food Anim Pract. 1997;13: 367–377. 9368983 10.1016/s0749-0720(15)30302-9

[10] Urban-Chmiel R, Grooms DL. Prevention and control of bovine respiratory disease. J Livestock Sci. 2012;3: 27–36.

[11] Hilton WM. BRD in 2014: where have we been, where are we now, and where do we want to go? Anim Health Res Rev. 2014;15:120–122. 10.1017/S1466252314000115 25358813

[12] Gorden PJ, Plummer P. Control, management, and prevention of bovine respiratory disease in dairy calves and cows. Vet Clin Food Anim. 2010;26: 243–259. 10.1016/j.cvfa.2010.03.004 20619182 PMC7135383

[13] Mosier D. Review of BRD pathogenesis: the old and the new. Anim Health Res Rev. 2014;15:166–168. 10.1017/S1466252314000176 25351390

[14] Mohanty SB, Lillie MG, Albert TF, Sass B. Experimental exposure of calves to a bovine rhinovirus. Am J Vet Res. 1969;30: 1105–1111. 4307580

[15] Ide PR, Darbyshire JH. Studies with a rhinovirus of bovine origin. Arch Gesamte Virusforsch. 1972;36: 335–342. 4336495

[16] Betts AO, Edington N, Jennings AR, Reed SE. Studies on a rhinovirus (EC11) derived from a calf. J Comp Path. 1971;81: 41–49. 4326296 10.1016/0021-9975(71)90053-3

[17] Rosenquist BD. Rhinoviruses: Isolation from cattle with acute respiratory disease. Am J Vet Res. 1971;32: 685–688. 4325245

[18] Kurogi H, Inaba Y, Goto Y, Takahashi A, Sato K. Isolation of rhinovirus from cattle in outbreaks of acute respiratory disease. Arch Gesamte Virusforsch. 1974;44: 215–226. 4365603 10.1007/BF01240609

[19] Collin EA, Sheng Z, Lang Y, Ma W, Hause BM, Li F. Co-circulation of two distinct genetic and antigenic lineages of proposed influenza D virus in cattle. J Virol. 2015;89:1036–1042. 10.1128/JVI.02718-14 25355894 PMC4300623

[20] Hause BM, Ducatez M, Collin EA, Ran Z, Liu R, Sheng Z, et al Isolation of a novel swine influenza virus from Oklahoma in 2011 which is distantly related to human influenza C viruses. PLoS Pathog 2013;9: e1003176 10.1371/journal.ppat.1003176 23408893 PMC3567177

[21] Neill JD, Bayles DO, Ridpath JF. Simultaneous rapid sequencing of multiple RNA genomes. J Virol Methods 2014;201: 68–72. 10.1016/j.jviromet.2014.02.016 24589514 PMC7119728

[22] Allander T, Tammi MT, Eriksson M, Bjerkner A, Tiveljung-Lindell A, Andersson B. Cloning of a human parvovirus by molecular screening of respiratory tract samples. Proc Natl Acad Sci. 2005;102: 12891–12896. 16118271 10.1073/pnas.0504666102 PMC1200281

[23] Tamura K, Stecher G, Peterson D, Filipski A, Kumar S. MEGA6: Molecular Evolutionary Genetics Analysis version 6.0. Molec Biol Evol. 2013;30: 2725–2729. 10.1093/molbev/mst197 24132122 PMC3840312

[24] Zhang X, Hasoksuz M, Spiro D, Halpin R, Wang S, Vlasova A, et al Quasispecies of bovine enteric and respiratory coronaviruses based on complete genome sequences and genetic changes after tissue culture adaptation. Virology 2007;363: 1–10. 17434558 10.1016/j.virol.2007.03.018 PMC7103286

[25] Dán Á, Ruzsics Z, Russell WC, Benkő M, Harrach B. Analysis of the hexon gene sequence of bovine adenovirus type 4 provides further support for a new adenovirus genus (Atadenovirus). J Gen Virol. 1998;79: 1453–1460. 9634088 10.1099/0022-1317-79-6-1453

[26] Palmenberg AC, Sgro J-Y. Topological organization of picornaviral genomes: statistical prediction of RNA structural signals. Semin Virol. 1997;8: 231–241.

[27] Pilipenko EV, Blinov VM, Dmitrieva TM, Agol VI. Conservation of the secondary structure elements of the 5’-untranslated region of cardio- and aphthovirus RNAs. Nucleic Acids Res. 1989;17: 5701–5711. 2548167 10.1093/nar/17.14.5701 PMC318190

[28] Stewart SR, Semler BL. RNA determinants of Picornavirus cap-independent translation initiation. Semin Virol. 1997;8: 242–255.

[29] Clarke BE, Sangar DV, Burroughs JN, Newton SE, Carroll AR, Rowlands DJ. Two initiation sites for foot-and-mouth disease virus polyprotein in vivo. J Gen Virol. 1985;66: 2615–2626. 2999308 10.1099/0022-1317-66-12-2615

[30] Hinton TM, Li F, Crabb BS. Internal ribosomal entry site-mediated translation initiation in equine rhinitis A virus: similarities to and differences from that of foot and mouth disease virus. J Virol. 2000;74: 11708–11716. 11090170 10.1128/jvi.74.24.11708-11716.2000 PMC112453

[31] Kozak M. The scanning model for translation: an update. J Cell Biol. 1989;108: 229–241. 2645293 10.1083/jcb.108.2.229 PMC2115416

[32] Lea S, Hernandez J, Blakemore W, Brocchi E, Curry S, Domingo E, et al The structure and antigenicity of a type C foot and mouth disease virus. Structure 1994;2: 123–139. 8081743 10.1016/s0969-2126(00)00014-9

[33] Thomas AA, Woortmeijer RJ, Puijk W, Barteling SJ. Antigenic sites on foot and mouth disease virus type A10. J Virol. 1988;62: 2782–2789. 2455819 10.1128/jvi.62.8.2782-2789.1988 PMC253712

[34] Medina M, Domingo E, Brangwyn JK, Belsham GJ. The two species of the foot and mouth disease virus leader protein, expressed individually, exhibit the same activities. Virology 1993;194: 355–359. 8386879 10.1006/viro.1993.1267

[35] Baxt B, Morgan DO, Robertson BH, Timpone CA. Epitopes on foot-and-mouth disease virus outer capsid protein VP1 involved in neutralization and cell attachment. J Virol. 1984;51: 298–305. 6205165 10.1128/jvi.51.2.298-305.1984 PMC254438

[36] Shan T, Li L, Simmonds P, Wang C, Moeser A, Delwart E. The fecal virome of pigs on a high-density farm. J Virol. 2011;85: 11697–11708. 10.1128/JVI.05217-11 21900163 PMC3209269

[37] Hause BM, Collin EA, Liu R, Huang B, Sheng Z, Lu W, et al Characterization of a novel influenza virus in cattle and swine: proposal for a new genus in the *Orthomyxoviridae* family. mBio 2014;5: e00031–14. 10.1128/mBio.00031-14 24595369 PMC3958797

[38] Bögel K. Bovine rhinoviruses. J Am Vet Med Assoc. 1968;152: 780–783.

[39] Ide PR, Darbyshire JH. Studies with a rhinovirus of bovine origin IV. Neutralizing activity against strain RS 3x in bovine sera. Arch Gesamte Virusforsch. 1972;36: 343–350. 4336496 10.1007/BF01249865

